# Removal of a Broken Cannulated Intramedullary Nail: Review of the Literature and a Case Report of a New Technique

**DOI:** 10.1155/2013/461703

**Published:** 2013-12-25

**Authors:** Amr A. Abdelgawad, Enes Kanlic

**Affiliations:** Department of Orthopedic Surgery, Paul L. Foster School of Medicine, Texas Tech University Health Science Center, 4801 Alberta Avenue, El Paso, TX 79905, USA

## Abstract

Nonunion of long bones fixed with nails may result in implant failure. Removal of a broken intramedullary nail may be a real challenge. Many methods have been described to allow for removal of the broken piece of the nail. In this paper, we are reviewing the different techniques to extract a broken nail, classifying them into different subsets, and describing a new technique that we used to remove a broken tibial nail with narrow canal. Eight different categories of implant removal methods were described, with different methods within each category. This classification is very comprehensive and was never described before. We described a new technique (hook captured in the medulla by flexible nail introduced from the locking hole) which is a valuable technique in cases of nail of a small diameter where other methods cannot be used because of the narrow canal of the nail. Our eight categories for broken nail removal methods simplify the concepts of nail removal and allow the surgeon to better plan for the removal procedure.

## 1. Introduction

In cases of nonunion of a fracture stabilized with intramedullary nails, fatigue failure of the nail can occur due to cyclic loading. Extraction of the broken nail will be needed in the revision surgery. Closed exchange nailing is becoming now one of the preferred methods to treat nonunion of fracture tibia and femur [1]. Closed removal of the broken distal nail can be a very hard task to achieve. Multiple techniques had been described to extract a broken distal piece of the nail, yet the procedure still can be very challenging and the commonly used methods and tools can fail to extract the broken part. In this paper, we are reviewing the different techniques to extract a broken nail, classifying them into different subsets, and describing a new technique that we used to remove a broken tibial nail with narrow canal. Pubmed and Google scholar search for words (broken nail, extraction, and removal) and cross-referencing were used to review the methods of nail extraction.

## 2. Case Report and Surgical Technique

Twenty-eight-year-old male had open tibial fracture. Patient was treated with vascularized flap for soft tissue coverage and intramedullary nailing (Stryker, Mahwah, NJ, USA). The nail was 9 mm in diameter. Six months later, patient had bone grafting for delayed union. One year after injury, patient presented with increased pain and deformity. Radiographs showed broken nail at the junction of the distal one-fourth with the proximal 3/4. Patient was taken to surgery for the removal of the nail, bone grafting, and introduction of thicker diameter nail.

The proximal part of the nail was removed easily using the universal extractor. The distal tip of the distal part of the nail did not accommodate the passage of two guide wires (Smith and Nephew, Memphis, TN, USA) to remove it using the stacking technique. Smaller guide wires from Synthes (Paoli, PA, USA) were tried with no success. Then we tried to use the distractor hook from Synthes. The hook was passed across the end of the nail, but it failed to hook and capture the nail. Multiple attempts were done to hook around the end of the nail; however, this was not successful. Trial to use the hook with a guide wire across the end of the nail (to allow the hook to better capture the end of the nail) was also not successful as the end of the nail did not accommodate the passage of both the guide and the hook.

We then used a new technique to make the hook stuck inside the distal piece of the nail and extract the piece by pulling the hook.

**Figure 1:**
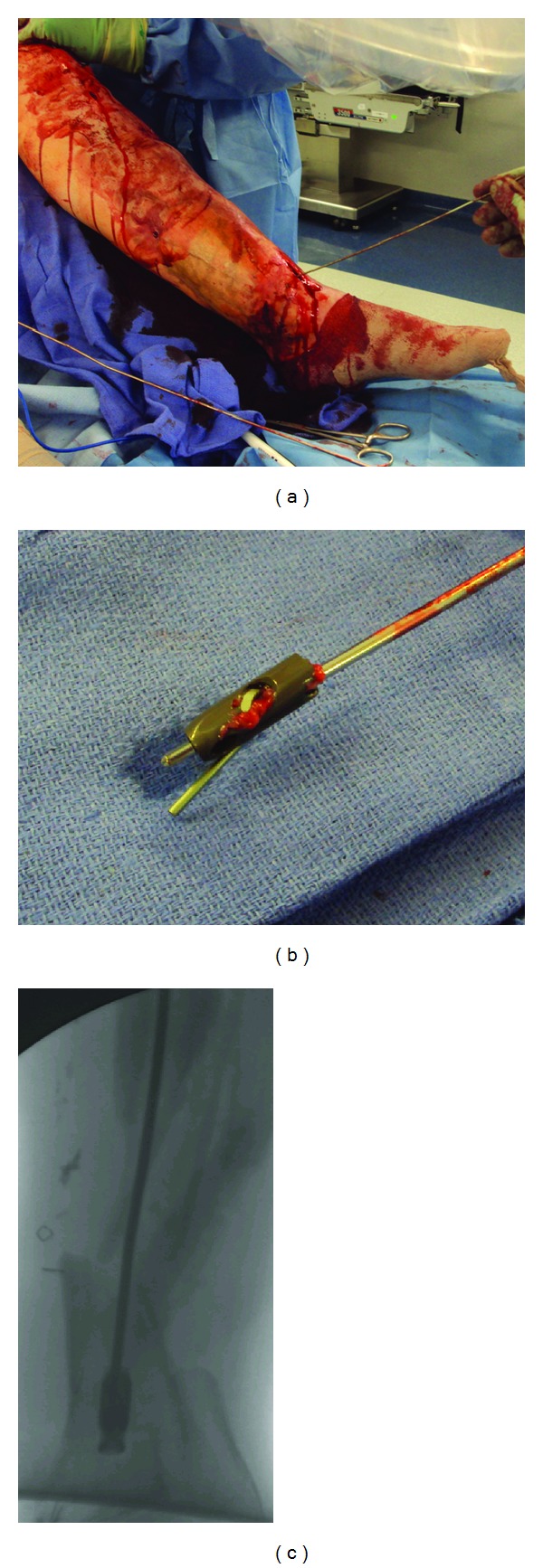
(a) Insertion of the flexible nail from the locking hole. The flexible nail was then cut at a distance of about 2 inches. (b) The broken piece was removed with the hook and the piece of the flexible nail wrapped around the hole of the locking screw. (c) Radiograph during extraction of the broken piece.

The hook was first introduced past the distal end of the broken piece. Then a small piece (around 2 inches) of flexible nail 2 mm was introduced into the slot of the distal locking screw. The hook was then pulled back and it became incarcerated inside the nail by the flexible nail piece. The hook was pulled out and because it finally became incarcerated inside the broken distal piece of the nail, the broken piece of the nail was pulled out (Figure 1).

## 3. Discussion

Various techniques have been described for the extraction of the distal piece of broken nail. Some of them require specific instruments; others use more generic tools that are readily available in most of the operating rooms. In general, regardless of the method used, good planning is of utmost importance to ensure that adequate and necessary tools are present during surgery. All efforts should be done to identify the brand, the size, and the manufacturer of the nail. If possible, obtaining a nail similar to the broken one may help the surgeon to know the inner diameter and to better plan for the removal.

### 3.1. Interference Fit Guide Wires

It is the most readily available method for surgeons and does not require any specific instrumentation.

**Figure 2:**
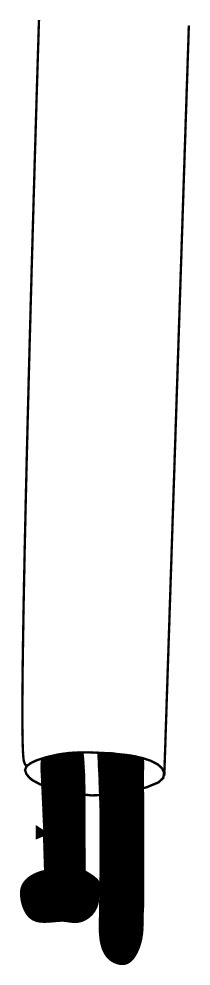
Interference fit guide wires. A non-tipped guide wire is passed beside a ball-tipped guide wire through the distal tip of the nail, and then the ball-tipped guide wire is pulled back to extract the nail.

Ball-tipped guide wire is passed through the distal nail tip; then another nontipped guide wire is passed across the nail tip (occasionally in large nails, 2-3 nontipped guide wire may have to be passed to gain good fit inside the nail outlet). Then the ball-tipped guide wire is pulled back to extract the nail [2, 3] (Figure 2). The end of the ball-tipped guide wire can be bent to increase its chance to pull the piece of the broken nail [4]. Karladani described a technique in which he used one guide wire that was trapped in the canal using a 3.5 mm screw inserted through the locking hole [5].

In our opinion, this may represent the most readily available method to extract a broken nail. In most cases it gives very predictable results. It is our first line method to remove a broken piece of cannulated intramedullary nail.

### 3.2. Hooks

#### 3.2.1. Hooks at the Distal End of the Nail

**Figure 3:**
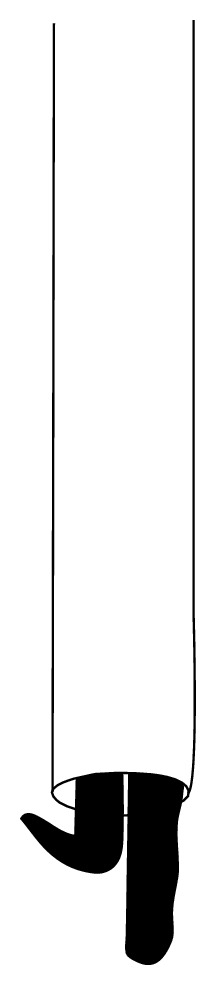
Special hook is passed beside another guide wire through the distal tip of the nail, and then the nail is removed by back extraction of the hook.

Special hooks are passed through the nail outlets and catch the end of the nail. Then the hooks are pulled back with the broken distal piece. This method had been widely used and many companies have their commercially available hooks [6, 7]. Often, another guide wire is needed to stack the hook to increase its chance in catching the end of the nail and pulling the nail with back extraction of the hook (Figure 3) [8]. Park et al. described a method of making a “groove” and a “bend” in a guide wire to act as a hook for use in nail with narrow diameter [9]. Acharya et al. described bending a guide wire to obtain a “fish hook” that is used to extract the nail by hooking into the end of the distal piece; the guide rod can be passed inside or outside the nail [10].

#### 3.2.2. Hook with Stacking from the Locking Hole

This is our new described modification for the hook use. In our case, the hook was not able to capture the outlet of the nail and we could not add another guide wire at the outlet due to the small size. In order to solve this problem and allow the hook to gain purchase in the nail, we used a flexible nail through the locking hole. The hook, the flexible nail, and the broken piece all were extracted as one unit. This method is useful in cases of narrow canals inside the nail that cannot accommodate two guide wires or hook and guide wire. Also due to this narrow canal, it was not possible to use “Karladani” technique [5] of a screw in the locking hole to get interference fit with the guide wire.

### 3.3. Press Fitting in the Hollow of the Nail

Various tools are used to gain purchase in the broken piece of the canal by press fitting in the hollow part of the nail. Commercially available conical threaded stainless steel extractors that can thread inside the nail are now available. This depends on the fact that most current nails are composed of titanium and aluminum alloys. The stainless steel tip on the extraction bit will displace the metal on the nail and have a wedge effect. The extractor can be pulled with the nail attached to it. Before threading the extractor in the nail, rotational stability of the broken piece has to be maintained. This is usually done by keeping the locking screws in place until the extractor is applied. If no locking screws are inserted in the broken piece, rotational stability can be achieved by a thick Steinman pin introduced in one of the locking holes [2].

Steinberg et al. described the use of Kuntscher nail to gain press fit in the broken distal piece of the nail [11]. The elastic open cross-section Kuntscher nail is forced into the nail fragment. Surgeon should be very cautious while trying to push the Kuntscher into the broken piece as this may require lots of force that can push the broken nail more distally. Also Kuntscher nail may not be available in most of the hospitals in North America nowadays. Sivananthan et al. used a nail 3 mm smaller than removed proximal piece of the nail to gain press fitting inside the broken piece [12].

Smith et al. described the use of 3.5 mm tap to grip in the cannulation of the nail [13]. Georgilas et al. used the reamer to wedge inside the broken piece of the canal. They used the extracted proximal piece as a guide for the size of the reamer used [14].

### 3.4. Removing the Broken Piece from the Opposite Side

#### 3.4.1. Femoral Nails

In cases of femoral nails, a hole can be made in the femoral notch (at the site of entry point of retrograde nails) to push the broken piece of nail retrograde to the greater trochanter entry site [15, 16]. Rather than pushing the nail into the knee, a guide wire can be introduced from the knee; the wire will engage into the nail and then the wire is pulled from the greater trochanter side [17]. Maini et al. used flexible nail instead of the guide wire with the flat side of the flexible nail acting as the “capturing” part that pulled the broken piece of the nail [18]. Magu used a washer over the guide wire to better capture the nail [19]. The reverse can be done in cases of broken retrograde femoral nail (extracting the nail from trochanteric area by pushing it from the knee) [20].

#### 3.4.2. Tibial Nails

Passing the guide wire from the broken end of the nail can also be done in the tibial side, however, this requires making a hole in the distal part of the tibia. Levine and Georgiadis described passing the guide wire from a small hall in medial malleolus to go through the distal outlet of the nail then through the knee entry hole. The guide wire (with the tip at the distal outlet) is pulled out from the knee with the broken piece. This technique may not be an easy one because of the angle that the guide wire has to capture the distal outlet [21].

### 3.5. Capture of the Nail from Outside

These methods may play more important role in solid nails that cannot be approached from within; however, they usually require specialized tools that are not readily available and significant overreaming of the medulla. The extractor is locked onto the nail by rotating the rachet grip until it is tight. This method is not commonly performed and requires reaming the medulla to about 4 mm more than the nail used which may be very hard to achieve or result in breaking of the bone [22, 23]. Gosling et al. described the use of the custom made extractor that goes over the nail and they are both connected by a narrow wire that is introduced through the holes in the extractor and the locking hole of the nail [24].

### 3.6. Bent Wire from the Locking Hole

A guide wire is bent, introduced beside the broken piece, and then turned to hook inside the looking hole of the nail. Because the nail is not pulled from the end, it may not stay in line with the axis of the medulla causing the nail to tilt and jam in its way out. To prevent this, a reduction tool was used over the guide wire and pushed against the nail [25].

### 3.7. Removal of the Nail through the Nonunion Site

The broken piece is directly pulled from the nonunion side by a hook or Kocher forceps [26, 27]. This technique will require marked stripping of the soft tissues and removal of some bone at the nonunion site to be able to remove the broken piece.

### 3.8. Removal of the Nail from a Hole in the Distal Metaphysis

A hole is made in the metaphysial part distal to the tip of the nail and the nail is first levered proximally with a Steinmann pin then pushed into the hole in the metaphysis using narrow-diameter nail inserted from above. A Hohmann-type retractor is inserted from the hole to guide the nail to outside [2, 28]. Considerable amount of bone has to be removed in this technique.

The techniques from Sections 3.5–3.8 can be used for solid nails.

In conclusion, the technique that we described (hook captured in the medulla by flexible nail introduced from the locking hole) is very valuable in cases of nail of a small diameter where other methods cannot be used because of the narrow canal of the nail. It is similar in principle to the technique described by Karladani using a guide wire and screw; however, our technique can be used in much small inner diameter of the nail. Our eight categories for broken nail removal methods simplify the concepts of nail removal and allow the surgeon to better plan for the removal procedure.
